# Evaluating the effectiveness of radiological anatomy course in a module of undergraduate medical curriculum

**DOI:** 10.12669/pjms.41.10.12623

**Published:** 2025-10

**Authors:** Naila Nadeem, Ambreen Surti, Azam Afzal, Mariyah Hidayat

**Affiliations:** 1 Naila Nadeem Associate Professor, Department of Radiology, Aga Khan University, Karachi, Pakistan; 2 Ambreen Surti Assistant Professor, Department of Biological and Biomedical Sciences, Aga Khan University, Karachi, Pakistan; 3 Azam Afzal Senior Instructor, Department of Educational Development, Aga Khan University, Karachi, Pakistan; 4 Mariyah Hidayat Professor, Department of Anatomy, University College of Medicine and Dentistry, University of Lahore, Pakistan

**Keywords:** Anatomy, Curriculum integration, Multiple-choice questions, MBBS curriculum, Radiology

## Abstract

**Objective:**

To evaluate the effectiveness of a Radiological Anatomy Module in the undergraduate MBBS curriculum

**Methodology:**

An experimental pre- and post-test study was conducted over a ten weeks period, from September 23, 2024 to December 2, 2024, at the University College of Medicine and Dentistry, University of Lahore. A total of 144 second year MBBS students were randomly assigned to either a control group or an experimental group. Both groups received equal teaching hours and were taught by the same trained facilitators using identical instructional methods-lectures, small group discussions, applied clinical scenarios and dissection. In addition, the experimental group was taught how to identify anatomical structures on radiological images, which were integrated into all teaching sessions. Assessments comprised 30 MCQs per module, validated by subject experts and subjected to item analysis. Student feedback was gathered through a structured questionnaire. Data were analyzed using SPSS version 25, with paired and independent t-tests applied (p < 0.05).

**Results:**

The experimental group demonstrated significantly higher post-test scores compared to the control group in both the Abdomen (20.5 ± 1.12 vs. 14.05 ± 1.53; p < 0.001) and Pelvis modules (23.7 ± 2.39 vs. 13.8 ± 1.42; p < 0.001), as determined by independent-sample t-tests. Item analysis confirmed that MCQs were of appropriate difficulty and discrimination, with acceptable reliability (Cronbach’s alpha: 0.61 for Abdomen, 0.59 for Pelvis). Feedback responses also indicated greater engagement, motivation and overall satisfaction among the experimental group (p < 0.001).

**Conclusion:**

Teaching abdominal and pelvic anatomy through the lens of radiology significantly improves understanding, reinforces clinical relevance and enhances student engagement.

## INTRODUCTION

In the evolving landscape of medical education, integrating radiology into undergraduate anatomy teaching is becoming increasingly important. Radiological imaging modalities such as X-rays, CT scans and MRIs provide dynamic, three-dimensional views of anatomical structures, helping students better understand complex spatial relationships and apply anatomical knowledge in clinical practice.^1^ Compared to traditional cadaveric dissection alone, radiological imaging offers an additional dimension to learning anatomy, preparing students for modern diagnostic work.^2^ Bringing radiology into medical teaching early allows students to connect anatomical theory with clinical scenarios and develop critical thinking and diagnostic reasoning skills.^3^ This approach fits well with the integrated modular curricula in medical schools, encouraging students to study basic and clinical sciences side by side. This approach also aligns with the principles of Behaviorist learning theory, which emphasize the role of repeated, reinforced exposure to relevant stimuli in shaping learning outcomes. By repeatedly associating anatomical structures with their radiological appearances, students receive immediate reinforcement that strengthens recall and facilitates the transfer of knowledge to clinical contexts.^4^ As technology advances, today’s doctors must be able to interpret and use diagnostic imaging confidently and building these skills should start early in medical education.^5^

Globally, many medical schools have begun integrating radiology earlier into their curricula to improve students’ clinical skills. In the UK, for example, the Royal College of Radiologists and the General Medical Council have recommended combining radiology with anatomy teaching to strengthen students’ diagnostic and clinical abilities.^6^ Evidence shows that merging radiological resources with traditional anatomy teaching leads to better student outcomes.^7^ Studies confirm that this integration improves students’ spatial understanding, clinical reasoning and readiness for modern healthcare practice.^8^

In Pakistan, however, this trend is yet to be fully embraced. The Pakistan Medical and Dental Council (PMDC) curriculum currently lacks formal guidelines on integrating radiology into anatomy teaching, resulting in inconsistent practices across medical colleges.^9^ However, there is growing awareness of the need to introduce radiological content formally into undergraduate anatomy education.^10^ Without this component, students may graduate without the imaging-related skills now expected in clinical practice. At the University of Lahore, internal curriculum reviews and student feedback have revealed a significant gap in the second-year MBBS curriculum-specifically, the lack of formal radiology instruction, which hinders students’ ability to correlate anatomical knowledge with clinical imaging. Despite using a variety of teaching strategies-interactive lectures, small group discussions, cadaveric dissection and anatomical models-students have repeatedly reported difficulties in applying anatomy to clinical practice, especially when interpreting diagnostic images. International studies further support these findings, demonstrating that integrating radiological anatomy enhances students’ understanding of spatial relationships and the clinical application of anatomical knowledge.^8^

Building on this gap, radiological integration offers a practical bridge between theoretical anatomy and clinical relevance. By familiarizing students with how anatomical structures appear in various imaging modalities (X-rays, CT, MRI and ultrasound), it fosters a more clinically oriented mindset early in training. Rather than solely relying on textbook visuals or cadaveric dissection, students benefit from a multimodal approach that develops spatial comprehension, diagnostic reasoning and the ability to apply anatomical knowledge in real-world scenarios. Studies have also associated this approach with improved academic outcomes and enhanced readiness for clinical responsibilities.^11^,^12^ At the University of Lahore, the introduction of a structured Radiological Anatomy Module presents a timely opportunity to align anatomy education with contemporary clinical and educational demands.

This initiative supports the broader goal of producing graduates who are well-equipped to interpret diagnostic imaging in clinical practice.^13^ In the existing MBBS curriculum at the University of Lahore, radiological anatomy is not formally introduced in the second year, creating a significant disconnect between theoretical anatomical knowledge and its clinical application. Both internal reviews and student feedback have repeatedly highlighted the difficulty students face in visualizing structures on imaging and applying anatomy in clinical settings. Internationally, integrated curricula have shown success in bridging this gap, yet structured evaluations of such interventions remain limited in Pakistan. This study addresses this curricular void by implementing and evaluating a structured Radiological Anatomy Module tailored to the local academic context. To assess the impact of this integration, the present study was designed with two key objectives: to evaluate the module’s effectiveness in improving students’ understanding and retention of anatomical concepts, as measured by pre- and post-test scores; and to gather students’ perceptions of the radiology-integrated curriculum through structured feedback. These objectives together aim to measure both the educational effectiveness and student satisfaction resulting from the integration.

## METHODOLOGY

This study employed an experimental pre- and post-test design involving control and experimental groups. Conducted at the University College of Medicine and Dentistry (UCMD), University of Lahore, the research targeted second year MBBS students enrolled in the ten weeks Abdomen and Pelvis module from September 23, 2024 to December 2, 2024. The Abdomen and Pelvis module was chosen due to its clinical relevance and complexity. At the time of the study, no formal radiological integration existed in the anatomy curriculum at UCMD.

### Ethical approval:

Administrative clearance from UCMD (Admin/UCM/1245) was obtained on September 16, 2024, followed by Ethical approval from Aga Khan University (2025-10649-32763), which was obtained on January 7, 2025, due to a delay in interinstitutional collaboration. Written informed consent was obtained from all participants. Confidentiality was maintained through anonymized coding and participation in the study was voluntary.

### Inclusion criteria:

- Participants included enrolled second-year MBBS students who had a minimum of 75% attendance, completed both pre- and post-tests and had no prior exposure to radiology-related instructional interventions.

### Exclusion criteria:

- Students with learning disabilities, health limitations effecting participation, or those who did not complete the study activities were excluded.

### Participants:

After applying the inclusion criteria, all 144 eligible second-year MBBS students were enrolled in the study. They were then randomly allocated into two equal groups of 72 each (Control and Experimental) using simple random sampling through a computer-generated random number list.

### Instructional Intervention:

The instructional phase was a multidisciplinary collaboration involving faculty from the Departments of Anatomy, Radiology and Medical Education. All participating faculty had at least five years of teaching experience and received structured pedagogical training through three 90-minute sessions conducted by the Department of Medical Education. These sessions focused on integrating radiological imaging into anatomy teaching, interpretation of imaging modalities (X-ray, CT, MRI) and facilitation techniques for interactive learning. Specifically, facilitation training included strategies such as using questioning techniques to stimulate critical thinking, promoting student-led discussions, managing small-group dynamics and employing case-based teaching approaches to engage learners in clinical reasoning. This ensured that the faculty were well-equipped to foster active participation, maintain student engagement and support collaborative learning during both traditional and radiology-integrated sessions. This training ensured instructional consistency and alignment across both study groups.

Students in the experimental group received integrated instruction combining gross anatomy with radiological imaging, using X-rays, CT, MRI and ultrasound alongside dissection. The control group received the same schedule of lectures and practical sessions, without radiological integration. In both groups, the same faculty facilitated instruction to minimize instructor bias. To equalize contact time, SGDs for both groups were extended by 15 minutes: with radiology-focused discussions for the experimental group and deeper anatomical reinforcement for the control group. This reinforcement involved a focused review of anatomical concepts using cadaveric prosections, anatomical models and textbook illustrations, encouraging students to clarify misconceptions, engage in peer teaching and apply anatomical knowledge to clinical case-based questions. Traditional cadaveric dissection was retained in both groups.

### Curriculum delivery:

In both modules, the control group followed the standard anatomy curriculum, which included interactive lectures supported by diagrams, anatomical models and cadaveric specimens; small group discussions on clinical cases without imaging; and dissection/prosection sessions. The experimental group underwent the same teaching schedule and was taught by the same facilitators but with radiological content embedded throughout. Imaging integration involved correlating anatomical structures with plain radiographs, ultrasound, CT and MRI during lectures and practical sessions, and linking dissection findings with relevant imaging to enhance spatial understanding and clinical application.

### Assessment design:

The intervention’s impact was measured using pre- and post-tests administered through UCMD’s e-assessment center. Each test included 30 rigorously developed multiple-choice questions (MCQs) per module, covering gross anatomy, clinical application and reasoning. To ensure assessment fairness between groups, no radiological images-such as X-rays, CT scans, MRIs, or ultrasound visuals-were included in the test items. However, during the teaching and learning phase, the experimental group was exposed to a variety of radiological resources, including labeled images, video clips of imaging procedures and live demonstrations, to enhance their understanding of anatomical structures. These visual tools were integral to the instructional strategy but were excluded from the formal assessment. The MCQs were collaboratively developed by faculty from Anatomy, Radiology and Medical Education and then reviewed by an expert panel, comprising two senior anatomists, one radiologist and one medical educationist with expertise in assessment design. The panel assessed the questions for content accuracy, clinical relevance and cognitive level. The items were piloted on a senior student cohort and following low initial reliability (Cronbach’s alpha < 0.5), the questions were revised and expanded to improve alignment with validated tables of specifications and Bloom’s taxonomy. Final reliability values improved to 0.61 for the abdomen module and 0.59 for the pelvis module.

### Data collection and analysis:

Both pre- and post-tests were administered at the beginning and end of each module, respectively, with randomized question in order to minimize recall bias. A structured, validated feedback questionnaire was distributed post-intervention to gather student perceptions. The questionnaire assessed three core domains: student engagement, instructional effectiveness and clinical relevance, each further divided into subthemes such as clarity of content, interactivity and overall satisfaction. Responses were recorded on a 5-point Likert scale ranging from ‘strongly disagree’ to ‘strongly agree.’

### Statistical analysis:

Data were analyzed using SPSS version 25. Paired t-tests assessed within-group changes, while independent t-tests compared between-group differences. A p-value < 0.05 was considered statistically significant. Descriptive statistics and Chi-square tests were used to analyze feedback. Reliability was confirmed using Cronbach’s alpha and content validity was ensured through expert alignment with intended learning outcomes.

## RESULTS

A total of 152 second-year MBBS students were initially enrolled in the Abdomen and Pelvis module at the University of Lahore. After applying predefined inclusion and exclusion criteria, 144 students were included in the study-72 each in the experimental and control groups. Eight students who did not meet study requirements or declined consent continued with standard instruction but were excluded from the research analysis. Baseline characteristics of both groups were comparable (Table-I), confirming that any differences in outcomes could be attributed to the instructional intervention.

**Table-I:** Baseline Characteristics of Study Participants.

Characteristic	Experimental Group (n=72)	Control Group (n=72)
Gender (M/F)	40/32	38/34
Mean Age (years)	21.3 ± 1.1	21.1 ± 1.3

To evaluate the quality of the MCQ-based assessments, an item analysis was conducted. Cronbach’s alpha values were 0.61 for the Abdomen module and 0.59 for the Pelvis module. While statistically significant differences were observed between the Abdomen and Pelvis modules in terms of difficulty and discrimination indices (Table-II), both modules demonstrated acceptable reliability, indicating that the assessments were appropriate for comparing instructional effectiveness.

**Table-II:** Comparative Item Analysis Metrics for Abdomen and Pelvis Modules.

Metric	Abdomen	Pelvis
Mean Difficulty Index	0.65	0.57
Mean Discrimination Index	0.45	0.52

Pre- and post-test comparisons demonstrated significant knowledge gains in both groups across both modules (Table-III). However, independent-sample t-tests confirmed that post-test scores were significantly higher in the experimental group for both modules (Table-IV), reflecting the positive impact of integrating radiological anatomy into teaching.

**Table III:** Comparison of Pre-Test and Post-Test Scores of both Modules.

Module	Group	Pre-Test (Mean ± SD)	Post-Test (Mean ± SD)	Mean Difference	p-value (within group)
Abdomen	Control	10.79 ± 1.84	14.05 ± 1.53	+3.26	<0.001
Abdomen	Experimental	12.50 ± 1.12	20.50 ± 1.12	+8.00	<0.001
Pelvis	Control	9.79 ± 1.38	13.80 ± 1.42	+4.01	0.002
Pelvis	Experimental	13.70 ± 1.09	23.70 ± 2.39	+10.00	<0.001

***Note:*** Paired t-test applied for within-group comparisons.

**Table IV:** Comparison of Post-test Scores between Control and Experimental groups in the Abdomen and Pelvis Modules.

Module	Group	Mean	SD	t-value (p)
Abdomen	Control	14.05	1.53	25.8 (0.001)
Abdomen	Experimental	20.50	1.12	
Pelvis	Control	13.80	1.42	30.05 (0.001)
Pelvis	Experimental	23.70	2.39	

***Note:*** The independent sample t-test determined statistical significance between groups and the p-value confirmed that the differences were unlikely to have occurred by chance.

Both groups demonstrated significant improvement following instruction; however, the greater magnitude of score increase in the experimental group indicates the added educational value of radiological integration.

Student feedback was strongly favorable toward the integrated approach (Table-V). Across all measured parameters-including engagement, motivation, clinical relevance, balance between theory and practice and overall satisfaction-the experimental group reported significantly more positive experiences compared to the control group.

**Table-V:** Student Feedback on Anatomy Learning Experience Across Both Groups.

Parameter	Experimental Group (n=72)	Control Group (n=72)	p-value
Sessions were engaging and interactive	63 (87%)	37 (52%)	<0.001
Improved motivation to study anatomy	60 (83%)	34 (47%)	<0.001
Helpful for clinical years	59 (82%)	35 (49%)	<0.001
Good mix of theoretical and practical teaching	58 (80%)	32 (44%)	<0.001
Overall satisfaction with learning experience	65 (90%)	38 (53%)	<0.001

***Note:*** Chi-square test applied for feedback comparisons.

## DISCUSSION

The present study demonstrated statistically significant improvement in post-test scores among students who received radiology-integrated instruction in the Abdomen and Pelvis module. Compared to the control group, the experimental group showed enhanced understanding of anatomical relationships, improved clinical reasoning and greater engagement with the content. As both groups were demographically similar with comparable baseline knowledge, the observed gains can be attributed to the instructional intervention.

These findings are consistent with international studies reporting the benefits of radiological integration in anatomy education. For example, Murphy et al. (2014) found that incorporating radiological images enhances spatial understanding and strengthens students’ clinical reasoning skills.^12^ Similarly, Estai and Bunt (2016) emphasized the effectiveness of multimodal teaching in improving comprehension of complex anatomical concepts.^13^ In line with this, Harden’s principles of integration support such interdisciplinary collaboration, encouraging the fusion of basic sciences with clinical application.^14^ Likewise, Evans and Watt (2005) in Australia, and Patel and Moxham (2006) in the UK, have underscored the importance of clinical imaging in modern anatomy education, noting its role in preparing students for diagnostic challenges.^15^,^16^

In addition, Triepels et al. (2020) found that the use of three-dimensional anatomy improved student understanding and supported their spatial comprehension.^17^ Moreover, Chau et al. (2025) reported improved preparedness for clinical decision-making among students exposed to radiology-enhanced teaching.^18^ Lufler et al. (2010) similarly found that incorporating radiology into anatomy curricula improves spatial understanding and student engagement.^19^ In the local context, on the contrary, structured evaluations of radiological integration at the undergraduate level in Pakistan remain scarce. One exception is Ara et al. (2021), who evaluated undergraduate students’ perceptions of radiology teaching within an integrated curriculum at Azad Jammu Kashmir Medical College.^20^ They found that a majority of participants supported its integration across the MBBS program, with 84% either agreeing or strongly agreeing to its inclusion from the first year onward.^17^

Mayer’s Cognitive Theory of Multimedia Learning explains why combining radiological images with narration/text improves spatial understanding and performance.^21^ In the present study, while both control and experimental groups improved after instruction, the experimental group achieved a markedly greater improvement, underscoring the positive impact of embedding radiological anatomy into the curriculum. Notably, students in our experimental group also demonstrated superior performance on clinical MCQs, despite the exclusion of radiological images from the assessment. This suggests that the intervention promoted transferable clinical reasoning skills. The effectiveness of applied clinical scenarios in health professional education has been widely recognized for enhancing diagnostic reasoning and application of knowledge.^22^ A related study by Afzal and Masroor (2019) further supports this, demonstrating improved engagement and learning outcomes in radiology through active learning strategies.^23^ Similarly, Rathan et al. (2022) at Gulf Medical University in the UAE incorporated radiological images into anatomy teaching and reported that imaging supported learning outcomes and maintained a manageable cognitive load, with over 80% of students expressing satisfaction.^24^ Although their approach was not identical to ours, both interventions highlight the value of integrating applied clinical contexts with radiological content to strengthen students’ ability to transfer anatomical knowledge to clinical problem-solving.

Student feedback in our study supports the effectiveness of integrating applied clinical scenarios with radiological content. The higher engagement, motivation and perceived clinical relevance reported by the experimental group are consistent with literature highlighting the value of contextual and clinically relevant learning environments in enhancing academic motivation and knowledge retention.^25^ These outcomes can be interpreted through the lens of Behaviorist learning theory,^4^ which emphasizes reinforcement through relevant and application-focused tasks, and Self-Determination Theory, which posits that meaningful learning experiences promote intrinsic motivation.^26^

The success of the intervention can also be attributed to the structured collaboration among faculty from the Departments of Anatomy, Radiology and Medical Education, which was central to achieving our objective of effectively integrating radiological content into the anatomy curriculum. This multidisciplinary approach ensured pedagogical consistency and clinical relevance, supporting the blended instructional model by integrating radiological content while preserving cadaveric dissection. This balance between modalities is consistent with behaviorist principles of reinforcement, and is supported by Dual Coding Theory, which highlights the benefits of pairing verbal and visual information to enhance cognitive processing.^27^ It is also consistent with recommendations by Harthoorn et al. (2024), who caution against completely replacing dissection with imaging technologies.^28^

### Strengths:

A key strength of this study is the use of a reliable and valid assessment tool, adding confidence to the results. The higher discrimination index in the experimental group indicates that radiology-integrated instruction more effectively differentiated student understanding. These findings demonstrate the educational impact of the intervention and support the value of theory-informed assessments in evaluating curricular innovations. Overall, the study adds evidence for integrating radiological anatomy in undergraduate medical education and underscores the potential of blended, interdisciplinary instruction to enhance comprehension, engagement and clinical readiness among medical students.

### Limitations:

This study was conducted at a single institution, which may limit generalizability to other medical colleges with different curricula and student demographics; however, it provides important context-specific evidence that can inform similar interventions in comparable settings. The relatively small sample size may have affected the statistical power and strength of conclusions, but it still yielded statistically significant and educationally meaningful differences between groups.

The study assessed only short-term knowledge acquisition without evaluating long-term retention or clinical application; nonetheless, measuring immediate learning outcomes was necessary as a first step before undertaking resource-intensive longitudinal research. The non-crossover design prevented within-subject comparisons, as only the experimental group received radiological integration during the study period; this design was chosen to prevent contamination between groups. The exclusive use of MCQs limited the assessment of higher-order cognitive skills such as clinical judgment and diagnostic reasoning; however, MCQs allowed for standardized, objective scoring across a large cohort, ensuring comparability between groups. Potential performance bias may exist due to faculty involvement in both instruction and evaluation, despite blinding measures; this was mitigated by using validated, pre-reviewed questions and consistent assessment protocols.

These limitations should be considered when interpreting the findings, which nevertheless contribute valuable evidence for radiology-integrated anatomy instruction in undergraduate medical education. In addition, the feasibility of implementing this intervention in low- and middle-income countries may be influenced by limited access to advanced imaging facilities, trained faculty and technical infrastructure. Successful replication would require faculty development programs focusing on radiological teaching skills and investment in basic imaging resources. Institutions with resource constraints may need to adopt phased or hybrid integration models to ensure sustainability.

## CONCLUSION

This study demonstrates that integrating a structured radiological anatomy module into the undergraduate anatomy curriculum improves student performance and engagement, while also receiving strong positive feedback. The findings indicate that such integration strengthens conceptual understanding, enhances clinical relevance and is well-received by students, supporting its value in anatomy education.
